# Evaluation of a breastfeeding promotion film among a racially minoritized sample

**DOI:** 10.1186/s12884-022-04607-0

**Published:** 2022-03-28

**Authors:** Kacie C. A. Blackman, Derek S. Slama, Trevor A. Pickering, Aqueelah Russell, Wenonah Valentine, Meridith A. Merchant, Carrie Saetermoe

**Affiliations:** 1 Health Sciences, Health Equity Research and Education (HERE) Center, California State University Northridge, Northridge, CA USA; 2 Population and Public Health Sciences, University of Southern California, Keck School of Medicine, Los Angeles, USA; 3 Antelope Valley Medical Center, Los Angeles, USA; 4 i.D.R.E.A.M. for Racial Health Equity, Los Angeles, USA; 5 Psychology, Health Equity Research and Education (HERE) Center, California State University Northridge, Northridge, CA USA

**Keywords:** Breastfeeding, Healthcare disparities, Black, Postnatal care, Health communications, Social support

## Abstract

**Background:**

In Los Angeles County (LAC), disparities in breastfeeding rates vary by race and region. Black persons are more affected by social and environmental factors than other racial/ethnic groups, leading to lower breast/chestfeeding rates. This study aims to evaluate the community’s knowledge, perceptions, experiences, barriers, and solutions before and after an educational film about Black persons who are breast/chestfeeding.

**Methods:**

Participant responses were collected anonymously through an online survey (via QR code) pre-and post-viewing a film with open- and closed-ended questions. There were 15 pre-screening questions and 24 post-screening questions discussed with a team of community experts. Questions included four main areas related to breast/chestfeeding: current/past experiences, support, awareness of laws, and solutions. Central tendency, variance, and paired differences were calculated from evaluation responses.

**Results:**

There were 185 participants who completed the pre-screening evaluation and 57 participants who completed the post-screening evaluation. Racial/ethnic differences were found for stated reasons for attendance, and perceptions of breastfeeding being challenging after viewing the video. On a five-point Likert scale (1 = very relevant, 5 = not relevant), most participants felt the video was relevant (median response = “2-relevant”; IQR = “3-neutral”; “1-very relevant”), learned something new (81.4%) and knew how to access breast/chestfeeding support after viewing the video (93.2%).

**Conclusions:**

Current media is a way to alter perceptions and opinions, and provides information. Additionally, it can be a way of increasing awareness of issues that Black breast/chestfeeding persons encounter. Strategic marketing efforts for future film screenings may increase attendance of those that can gain insight into breast/chestfeeding support (youth/young adults and males). Supportive breast/chestfeeding environments can also be a reality with readily accessible, unified, and encouraging personal and professional networks.

## Introduction

Breastfeeding has long stood as a protective factor against many illnesses including asthma, diabetes, and sudden infant death syndrome (SIDS) [1]. The Centers for Disease Control and Prevention (CDC) estimates that over 84.0% of infants born in the United States in 2017 are breastfed at some point in their infancy [2]. However, Black infants are significantly less likely to be breastfed at any point than any other racial or ethnic group [3]. They are also 2.3 times more likely to die than non-Hispanic white infants, specifically with preventable diseases such as respiratory infections, diarrheal diseases, and SIDS [4]. Breastfeeding can serve as a protective mechanism that can reduce the risk of preventable diseases that Black infants are highly susceptible to.

The socio-cultural, structural, and historical factors that influence the likelihood of breastfeeding for Black persons are complex. Low numbers of Black persons who breastfeed can be attributed to historical trauma of slavery-era practices of wet-nursing and “mammy” stereotypes [5]. In addition, research shows that caesarian section (planned or emergency) is a predictor of a lesser likelihood to breastfeed because of reduced opportunity to receive skin-to-skin contact immediately after delivery, and stress responses that can affect one’s ability to consciously focus on infant latching which reduces their ability to induce lactation [6]. In 2016, in Los Angeles County (LAC) Black persons (39.0%) had a higher caesarian section proportion compared to the county average (33.0%) and to their counterparts [7]. Further, socio-cultural and interpersonal stressors impact allostatic load of Black birthing persons, which in turn lead to lower birth weight or preterm birth (longer hospital stays) among Black infants compared to their counterparts; thus resulting in a lower likelihood of exclusive breastfeeding [7]. Such socio-cultural and structural factors demonstrate the need for protective, supportive, and respectful care alongside health communication platforms to support and promote healthy babies and breastfeeding among Black persons. Additionally, promotion of cultural responsiveness and increased awareness of implicit bias to reduce interpersonal racism in health care and social service settings are essential.

The purpose of this evaluation was to assess the community’s knowledge, perceptions, experience, barriers, and solutions regarding breastfeeding before and after viewing a culturally informed film.

## Methods

### Study setting

There were five film screenings held in four different geographic areas in LAC in August 2019. Each screening involved viewing a 90-min documentary film focusing on the influence of race on breastfeeding rates and the many challenges of three families’ experiences. All in-person film screenings were located at community-based/educational settings including one church, university, county-operated health clinic, public library, and medical treatment center. To ensure representation, the collaborators of the screenings (BreastfeedLA, i.D.R.E.A.M. for Racial Health Equity, Mocha Moms (San Fernando Valley and Los Angeles Chapters), CinnaMoms (Public Health Foundations Enterprises Women, Infants, and Children (WIC)), South Los Angeles Health Projects-WIC, California State University Northridge (CSUN) Institute of Community Health and Wellbeing, CSUN Magaram Center)) selected the five sites across four geographic locations including one each in west Los Angeles, northeast and northwest Los Angeles, as well as two sites in south Los Angeles.

#### Participants

Each screening was open to the public, and was advertised via collaborators, social media, and word of mouth. Participants were required to register through an event website in order to attend. Participants were given the QR code for pre- and post-screening survey participation if they were at least 18 years of age and had attended at least one of the film screenings. The group of people who completed the post-survey was a subset of the group that completed the pre-survey. Respondents gave their informed consent to participate. Before completing an online pre- or post-survey, participants were advised on the anonymity and confidentiality of all responses.

### Ethical considerations

This study was planned with the ethical principles of the Belmont Report in mind. We attended to respect for all people by intentionally building a broad base of participants. We are beneficent in our goal to inform Black persons about breast/chestfeeding and to normalize a healthy behavior that leads to health equity for Black infants and mothers (justice). Ethical principles of community engagement permeated our work; each phase of our work was informed and largely directed by Black persons in affected communities. Respondents gave their informed consent to participate. The Institutional Review Board of California State University Northridge (registry number: IRB-FY20-414) approved the study.

### Film 

Produced by Elizabeth Bayne, MFA and MPH*, Chocolate Milk: The Documentary* [8] was developed to build awareness and inspiration around Black women breastfeeding. By describing the historical and structural reasons why Black women do not breastfeed, the film follows three lactating Black women over the course of a year. This study examines the impact of the film on awareness and understanding of the barriers and joys of breastfeeding for Black women to suggest ways to address the structural barriers that discourage Black women from breastfeeding.

### Study design

Each location involved a pre- and post-screening survey provided at the beginning and end of each 90-min film screening. All entries were completed electronically by utilizing the QR code function on iPhones, iPads, or Android devices at the beginning and end of each screening. A link to the pre-screening survey was also sent through an email reminder prior to the screening, while a link to the post-screening survey was sent through a follow-up email. The lead author (KB) collected data. The data were submitted electronically and the lead author (KB) downloaded and assembled the data for cleaning and screening.

### Pre-screening survey

There was a total of 15 pre-screening survey questions. Pre-screening surveys identified sociodemographic factors including age, race, ethnicity, gender, residential zip code, occupation, and reasons for attending the screening. Race/ethnicity was classified into 1) whether the participant reported “Black” as one of their racial identities and 2) whether the participant indicated they were of Hispanic identity. Supplemental pre-screening questions were developed by study authors (KB, WV, MAM), which were based on previous maternal and infant health community-led programs designed and implemented by i.D.R.E.A.M. for Racial Health Equity. Items were selected for relevance to the film and included: breast/chestfeeding knowledge, motivation, experiences of those attending to bridge their lived experiences and how the screening may apply to them or the communities they serve. Participants were asked about their motivations for attending the screening, why they came, and at which location.

Additional questions queried broader knowledge of breastfeeding rights. Participants were asked, “Are you aware of the breastfeeding protection laws in California? (Yes/No)” for contextual understanding in background knowledge each participant had prior to viewing the film. Participants were then asked about their own experiences of personal breastfeeding and pumping history to understand personal experiences, strengths, and challenges that may influence participation in the film viewing: “Are you currently breastfeeding or pumping your milk for your baby? (Yes/No)”. In questions pertaining to breastfeeding experiences such as “Did you ever breastfeed or pump milk for your babies?”, an option of “not applicable” was provided, where appropriate, for those who may not have birthed a child or had an opportunity to breastfeed but attended the screening.

### Post-screening survey

There was a total of 24 post-screening survey questions. At the end of each screening and similar to the pre-screening survey, participants were asked about race, ethnicity, gender, age, residential zip code, and the location in which they viewed the film. The remaining questions explored perceptions about whether the film imparted any new knowledge to the viewers, what their views on breastfeeding in the Black community were, and what viewers’ initial reactions to the film were (e.g., “After watching the film, what surprised you the most?”).

Next participants were asked similar questions to the pre-screening survey (Table 1) as well as new questions surrounding their personal breastfeeding and pumping history which included seven questions and three secondary questions. Participants were asked about significant barriers/challenges they or their loved ones have faced when breastfeeding along with strategies to overcome such barriers and challenges, knowledge of how to access breastfeeding support, and the best way to create a supportive environment for breastfeeding. Two questions were on a five-point Likert scale: “Please rate the film’s relevance to your or your loved one’s breastfeeding experience” (1 = very relevant, 5 = not relevant at all); “If you have breastfed, or currently breastfeed, please rate on a scale of 1 to 5 how challenging breastfeeding was or is for you?” (1 = extremely challenging, 5 = no challenges).

**Table 1:** Survey topics

Survey Topics	Pre and/or Post
Motivations for attending the film screening	Pre
Hear about this event?	Pre
Breastfeeding protection laws awareness	Pre
Breastfeed or Express Milk history	Pre and Post
Most surprising film content	Post
Anything new learned?	Post
Views on breastfeeding in the Black community	Post
Current breastfeeding or milk expression	Post
Barriers and challenges with breastfeeding	Post
Solutions to breastfeeding barriers and challenges	Post
Access to breastfeeding support	Post
Film’s relevance to personal breastfeeding experience	Post
Supportive Breastfeeding Environment Features	Post

### Statistical analysis

Data analysis was completed by study authors (KB, DS, TP). Descriptive statistics including mean (standard deviation) and median (interquartile range—IQR) were computed on all pre-screening and post-screening survey questions, including demographic questions such as race/ethnicity, age, occupation, and residential zip code. To examine differing effects for Black vs. non-Black participants, survey responses were compared between these two racial groups with chi-square or Wilcoxon tests, as appropriate. In the post-screening survey, the following survey responses were reverse coded: 1 = “not relevant”/ “no challenges”, 5 = “very relevant”/ “extremely challenging”. Analyses were computed in R (v3.6.3), including the use of the *gtsummary* package.

## Results

There were 389 people who registered for the screenings, with 278 participants attending (assessed via sign-in sheets). Participants resided in 115 different LAC zip codes (22.8% of all LAC zip codes). Over 95% of respondents identified as female. The median age of participants completing the pre-survey was 38 (IQR = [29, 48], *n* = 182), while the median age of participants completing the post-survey was 37 (IQR = [25.2, 50.5], *n* = 54). For occupation, a third of respondents selected health professional (32.0%), followed by community member (29.0%), followed by student (20.0%), and agency staff (19.0%). Most of the attendees who were survey respondents attended the university location film screening (27.1%) and the location with the lowest number of respondents was the church location (13.3%).

Table 2 includes the proportion of race of those who self-identified on each pre- and post-screening survey. The highest representation across all locations was Black/African American (41.6%) in the pre-screening survey and both Black/African American and White (28.1%) in the post-screening survey. Nearly 16.0% of respondents did not mark race in the post-screening survey.

**Table 2:** Respondents by race/ethnicity

Race	Number of Respondents	
	Pre-Screening (*n* = 185)	Post-Screening (*n* = 57)
Black/African American	77 (41.6%)	16 (28.1%)
Latinx	20 (10.8%)	8 (14.0%)
White	44 (23.9%)	16 (28.1%)
Multiracial	15 (8.1%)	5 (8.8%)
Asian American/Pacific Islander	11 (5.9%)	2 (3.5%)
Native American	6 (3.2%)	1 (1.8%)
Other	12 (6.5%)	0 (0.0%)
Did Not Mark Race	0 (0.0%)	9 (15.8%)

### Pre-screening survey results

A total of 185 pre-screening evaluations were completed across all locations. Pre-screening responses ranged from 28 to 55 surveys at each location with the university location having the largest number of pre-screening responses.

Motivations for attending the workshop appeared to vary by race (Table 3). Overall, 36.6% of participants reported wanting to attend because they thought the workshop would be interesting. However, Black participants were more likely than non-Black participants to report that they wanted to attend due to experiencing barriers with breastfeeding and/or breastfeeding support (36% vs. 25%, *p* = 0.088) and due to wanting the opportunity to network (43% vs. 27%, *p* = 0.021). Black participants were less likely to report wanting to attend because they desired to learn more about breastfeeding support (35% vs. 73%, *p* < 0.001). Additionally (not shown in table), Hispanic participants were more likely than non-Hispanic participants to want to learn more about breastfeeding support (71% vs. 50%, *p* = 0.007) and less likely to want an opportunity to network (22% vs. 40%, *p* = 0.012).

**Table 3:** Pre-screening responses

**Characteristic**	**Overall**, *N* = 186^a^	**Black**, *N* = 77^a^	**Not Black**, *N* = 109^a^	***p*-value**^b^
**Breastfeeding Experience**				
Ever breastfed	111/125 (88.8%)	57/61 (93.4%)	54/64 (84.4%)	0.11
Number breastfed	2 (1, 2)	2 (1, 2)	2 (1, 2)	0.70
Experience breastfeeding (months)	13 (8, 27)	13 (9, 24)	13.5 (8, 30)	0.60
Aware of breastfeeding protection laws	125/186 (67.2%)	47/77 (61.0%)	78/109 (71.6%)	0.13
**Stated Reasons for Attendance**				
It sounded interesting	68 (36.6%)	30 (39.0%)	38 (34.9%)	0.60
Previously experienced breastfeeding barriers	55 (29.6%)	28 (36.4%)	27 (24.8%)	0.088
To learn more about breastfeeding	107 (57.5%)	27 (35.1%)	80 (73.4%)	< 0.001
For networking opportunities	62 (33.3%)	33 (42.9%)	29 (26.6%)	0.021

^a^n (%); Median (IQR)

^b^Pearson's Chi-squared test, Wilcoxon rank sum test

While not statistically significant, Black vs. non-Black (61% vs. 72%, *p* = 0.13) and Hispanic vs. non-Hispanic (62% vs. 70%, *p* = 0.20) participants were less likely to be aware of breastfeeding laws. Compared to non-Black participants, Black participants were more likely to have ever breastfed a child (93% vs. 84%, *p* = 0.11) while Hispanic participants were less likely to have ever breastfed a child compared to non-Hispanic participants (81% vs. 91%, *p* = 0.11).

### Post-screening survey results

A total of 57 post-screening evaluations were completed across all locations. The university location had the highest number of responses (56.0%). Table 4 lists the post-screening responses. The majority of respondents (81.4%) stated that they had learned something new from viewing the film; this proportion did not vary by race or ethnicity. Almost two-thirds of respondents (63%) reported changing their views on breastfeeding in the Black community.

**Table 4:** Post-screening survey results

**Characteristic**	**Overall**, *N* = 55^a^	**Hispanic**, *N* = 27^a^	**Not Hispanic**, *N* = 28^a^	***p*-value**^b^	**Black**, *N* = 17^a^	**Not Black**, *N* = 31^a^	***p*-value**^b^
**Skills**							
Learned something new	35/43 (81.4%)	16/18 (88.9%)	19/25 (76.0%)	0.40	13/16 (80.5%)	20/25 (80.0%)	> 0.90
Previously accessed breastfeeding support	28/37 (62.2%)	6/13 (46.2%)	17/24 (70.8%)	0.14	9/15 (60.0%)	13/19 (68.4%)	0.70
Now know how to access support or who to contact	41/44 (93.2%)	15/17 (88.2%)	26/27 (96.3%)	0.50	16/17 (94.1%)	23/25 (92.0%)	> 0.90
**Attitudes**							
Changed views on breastfeeding in the Black community	29/46 (63.0%)	16/19 (84.2%)	13/27 (48.1%)	0.013	10/17 (58.8%)	16/26 (61.5%)	0.90
Was previously unaware of breastfeeding issues in the Black community	5/29 (17.2%)	5/16 (31.2%)	0/13 (0.0%)	0.048	0/10 (0%)	5/16 (31.2%)	0.12
**Ratings**							
Rate the film’s relevance	4.0 (3.0, 5.0)	4.0 (3.0, 4.0)	5.0 (3.0, 5.0)	0.057	5.0 (13.75, 5)	4.0 (3.0, 4.5)	0.12
Rate how challenging breastfeeding is (for current/former breast feeders)	2.5 (2.0, 3.0)	3.0 (3.0, 3.0)	2.0 (1.0, 3.0)	0.04	2.0 (1.0, 2.0)	3.0 (2.0, 3.25)	0.007

^a^n (%); Median (IQR)

^b^Fisher’s exact test; Pearson's Chi-squared test; Wilcoxon rank sum test

While this proportion did not differ by Black vs. non-Black race, Hispanic individuals were more likely to report changing their views on breastfeeding in the Black community (84% vs. 48%, *p* = 0.013). When asked about how the film changed their beliefs (open-ended), 16% of respondents reported a theme of being unaware of the issues Black mothers face (28% in non-Black participants vs. 0% in Black participants, *p* = 0.03).

Black participants reported finding the film more relevant than non-Black participants (median = 5, IQR = [5,3.75] vs. median = 4, IQR = [3, 4.5], respectively, *p* = 0.12). Hispanic participants reported the film as less relevant to them vs. non-Hispanic participants (median = 4, IQR = [3, 4] vs. median = 5, IQR = [3, 5], respectively, *p* = 0.06).

Black participants had lower median ratings of finding breastfeeding challenging (median = 2, IQR = 1,2) compared to non-Black participants (median = 3, IQR = 2, 3.25, *p* = 0.007). Hispanic participants had higher median ratings of finding breastfeeding challenging (median = 3, IQR = 3,3) vs. non-Hispanic participants (median = 2, IQR = 2,3, *p* = 0.04).

Common challenges of breastfeeding faced by respondents included: latching on, baby nursing only on one side, time and experience, school and work challenges, inducing lactation with adopted children, knowing when to ask for support and where to find support, family is uncomfortable with breastfeeding in public or in front of men/male spouse, family not understanding reason for breastfeeding, or spousal abandonment.

Common solutions to increase breastfeeding reported by respondents included: reading books, lactation support groups, lactation educator/consultant, doula, partner, family, using an adequate pump effectively and often, patience with self, breastfeeding support from professors, breastfeeding support at work, supplemental nursing system, and pediatrician.

Table 5 describes new concepts reported (e.g., body awareness) and the supportive breastfeeding environment themes which included breastfeeding as a norm, availability and ambience of lactation space, community awareness, providing assistance, encouragement, additional support, access to affordable postpartum classes, male support, and support from those not interested in personally breastfeeding.

**Table 5:** Qualitative responses

New Concepts Learned	Example Quotes
Lactation Support	“How much extra support the Black community needs to normalize and destigmatize breastfeeding.”
	“I was most surprised about how difficult it is to get information or assistance, even for women who really want to breastfeed.”
Professionalism and Medical professionals	“How unprofessional and unsupportive doctors can be about breastfeeding.”
Lactation Profession Education	“New requirements were added for IBCLC which can definitely be a barrier for lactation educators of color.”
White privilege/Systemic	“How the midwife was treated when she opened her own birth center. I shouldn’t be surprised, but as a white person I’m often not aware of when or how these things happen. Which is why I came today, to learn.”
	“Culturally breastfeeding is seen as a white privilege.”
	“Breastfeeding support stems from systemic barriers.”
Health Statistics	“I knew some of the statistics but to hear all of them listed and to hear them coming from Black sisters was just so powerful and spoke loud and clear on so many levels.”
Body Awareness	“I learned that skin to skin can introduce and prompt breastfeeding.”
**Supportive Environment Themes**	
Breastfeeding as a Norm	“Unspoken. Where breastfeeding is the norm and other topics are conversed, such as business, traveling, daily routines, etc. life goes on.”
Availability and Ambience of Lactation Space	“Every building has a mother’s room…a low lit, climate controlled room with a clean bed or sofa, with the sounds of trickling water or white noise, to comfortably feed my baby.”
Community Awareness, Providing Assistance, or Encouragement	“One where people encourage you to breastfeed and help accommodate your breastfeeding needs.”
	“Having open conversations about the benefits of breastmilk and honestly just them seeing me openly breastfeed and the development of my daughter.”
Additional Support	“Meal train.”
	“Job that gives me space and time to pump. Paid parental leave.”
Access to Affordable Postpartum Classes	“…Ongoing support groups for postpartum care (health professionals; peers)”
Male Support	“…Men who proudly brag about the women in their lives breastfeeding.”
Support from Those Not Interested in Personally Breastfeeding	“…Those who are not interested gain insight and knowledge on breastfeeding and ways to be supportive.”

## Discussion

The study revealed many themes that reflect community perceptions and knowledge of breastfeeding challenges and needs for Black persons. First, many respondents were very surprised at the limitations and challenges not only for Black parents, but the low number of Black midwives available to the community. Black persons providing services to Black families is essential for cultural congruency and cultural connection as this allows for such midwives, doulas, or lactation specialists to act as a community leader for establishing trust that is based in cultural knowledge and respect. Similarly, the lack of equitable pay for Black midwives, doulas and lactation specialists in LAC exemplifies the deep-rooted effects of systemic and structural barriers, calling on the need to address them as a priority course of action (e.g., local budgeting and legislation, health plan coverage).

Participants also expressed the need for social support, particularly from family, friends, and other systems such as at work and/or school. For Black communities, family acts as an educator (communalism, a component of Black culture and collective identity) and this connection is essential [9]. Addressing subjective norms within families is especially important when reducing stigmatization for breastfeeding among Black persons. This includes providing opportunities for partners (e.g., medical office visits, in-hospital lactation support) to be involved in the breastfeeding experience to understand the importance of breastfeeding and how to provide support. Additionally, there is a need for paid parental leave that provides a livable wage to low and middle-income families (currently replaces 60% of wages) (CA AB 123) [10] so that breastfeeding can be done in a nurturing environment. The amendment to this bill (replaces 90% of wages) was passed (September 08, 2021) after the collection of data had ended. Further, high schools, higher education institutions, and workplaces need to support breastfeeding and enforce protections for lactating persons (e.g., students, employees, visitors). For example, it is of critical importance to have lactation education requirements during high school and college first-year, to have ample time between classes so that lactating students can express and store breast milk (~ 30–45 min) and improve visibility and quality of lactation spaces on campus.

Barriers to accessing breastfeeding support was a significant theme that emerged. Less than half of respondents shared that they knew where to access breastfeeding support. This highlights the need for equitable access and distribution of resources as well as organizations that create a sense of belonging that would attract Black persons. Many participants also shared that a supportive breastfeeding environment would include access to affordable postpartum classes (e.g., virtual, webinars, social media, in-person, at high school and higher education, workplaces, community centers, local parks, shopping malls, festivals, health fairs), indicating a need for more quality and culturally informed breastfeeding services. In addition to postpartum classes, preconception and prenatal education are important times to provide culturally responsive education and support all involved family members.

### Limitations

Overall, lower participation in the post-test presents a challenge to generalization. Since most participants were referred to the film viewings from an organization, many other Black persons who are not connected with an organization may need breastfeeding support yet not connect to services. Therefore, efforts are needed for identifying strategies to engage a broader representation of the Black population to build self-efficacy and reduce stigma on breastfeeding. It is likely that social media (Instagram, Twitter, TicTok, Facebook) are strong avenues to reach individuals who are not connected with organizations.

Because 95% of our sample were women, there is a need for more representation of non-women/non-females, males, older adults and youth/young adults to view Black breastfeeding promotion media. We acknowledge that there was a low post-survey response rate including proportionally fewer Black participants that answered the post-survey. This highlights a need for event hosts and evaluation team to have a more concerted effort for obtaining post-screening surveys (e.g., incentives, email/phone call follow-up).

### Future directions

Participants identified strategies that could be helpful in engaging the Black community in breastfeeding. It is important to inquire within this community, actively listen, and include them in collective thought, decision-making, and sustainable action. Some of the strategies included culturally congruent, high-quality and accessible community and family support, and job support in lactation accommodations. These efforts could be carried out by local non-profit organizations, community members, health professionals, and county-wide initiatives. For future iterations, it will be important to recruit Black parents who have never breastfed or who do not have intentions to breastfeed. Last, it is critical to inform all people, especially community members and students about breastfeeding laws.

## Conclusion

Media can be an effective way to alter perceptions and opinions on breastfeeding in the Black community. Enhancing marketing efforts for future film screenings may increase attendance of those who would gain insight into breastfeeding support (youth/young adults, non-women/non-females, older adults, and males). Supportive breastfeeding environments can be a reality with a readily accessible, unified, and encouraging team of personal and professional networks.

Health communication strategies can be effective in building self-efficacy for both the act of breastfeeding and accessing services, especially long before giving birth. Media can be used to emphasize the importance of breastfeeding and aid in modeling breastfeeding for Black parents and those who support them to reduce associated stigma created from intergenerational trauma. Media platforms include social media, podcasts, local news, television, advertisements and pictures that make the breastfeeding experience more visible. It is paramount to engage media professionals that have a strength in messaging to translate research findings to palatable and meaningful experiences that reflect familiarity and experience with the Black community. There is also a need for cultural engagement and responsiveness integrated into patient care and improvement in representation in health care to provide holistic and culturally tailored lactation supports from lactation consultants, doulas, midwives, perinatal professionals, and pediatricians.

One way to build health and social equity for Black families is to include their voices and priorities in a strong, loving, and joyous practice of breastfeeding. Healthy physically, emotionally, socially, and spiritually, breastfeeding can fortify and build resilience in Black families, building warmth, health, and mutual support as a unit. Representations of healthy lactating Black families, along with strong data supporting breastfeeding, can strengthen Black families, communities, and babies.

Collectively, perinatal care professionals must consistently follow-up with pregnant and lactating persons and link them to affordable, trustworthy, useful, and culturally-informed supports via various formats (e.g., digital, in-person, phone). These conversations can decrease risk of biased misinformation and strengthen client-centered informed decision-making. These efforts may assist in birthing and lactating persons feeling more supported and potentially increase intentions and breastfeeding outcomes (initiation, continuation), practices that lead to greater health equity while saving Black lives. Films that depict the joys and challenges of breast/chestfeeding can make deep social change by humanizing breast/chestfeeding while revealing historical and structural inequities that interrupt healthy family processes. Black families who are informed and proud of breast/chestfeeding can become a norm that has a profoundly positive impact on infant and family health and wellbeing.

## Data Availability

The dataset generated and analyzed during the current study are not publicly available due to the non-profit organization collaborators storing the data but are available from the corresponding author on reasonable request.
